# Somatic Mutagenesis with a *Sleeping Beauty* Transposon System Leads to Solid Tumor Formation in Zebrafish

**DOI:** 10.1371/journal.pone.0018826

**Published:** 2011-04-21

**Authors:** Maura McGrail, Julia M. Hatler, Xianyan Kuang, Hsin-Kai Liao, Kishore Nannapaneni, Kristin E. Noack Watt, Juli D. Uhl, David A. Largaespada, Erik Vollbrecht, Todd E. Scheetz, Adam J. Dupuy, Jesse M. Hostetter, Jeffrey J. Essner

**Affiliations:** 1 Department of Genetics, Development and Cell Biology, Iowa State University, Ames, Iowa, United States of America; 2 Department of Genetics, Cell Biology and Development, Masonic Cancer Center, Center for Genome Engineering, University of Minnesota, Minneapolis, Minnesota, United States of America; 3 Departments of Ophthalmology and Biomedical Engineering, University of Iowa, Iowa City, Iowa, United States of America; 4 Department of Anatomy and Cell Biology, University of Iowa, Iowa City, Iowa, United States of America; 5 Department of Veterinary Pathology, Iowa State University, Ames, Iowa, United States of America; University of Birmingham, United Kingdom

## Abstract

Large-scale sequencing of human cancer genomes and mouse transposon-induced tumors has identified a vast number of genes mutated in different cancers. One of the outstanding challenges in this field is to determine which genes, when mutated, contribute to cellular transformation and tumor progression. To identify new and conserved genes that drive tumorigenesis we have developed a novel cancer model in a distantly related vertebrate species, the zebrafish, *Danio rerio*. The *Sleeping Beauty* (*SB*) *T2/Onc* transposon system was adapted for somatic mutagenesis in zebrafish. The carp ß-actin promoter was cloned into *T2/Onc* to create *T2/OncZ*. Two transgenic zebrafish lines that contain large concatemers of *T2/OncZ* were isolated by injection of linear DNA into the zebrafish embryo. The *T2/OncZ* transposons were mobilized throughout the zebrafish genome from the transgene array by injecting SB11 transposase RNA at the 1-cell stage. Alternatively, the *T2/OncZ* zebrafish were crossed to a transgenic line that constitutively expresses SB11 transposase. *T2/OncZ* transposon integration sites were cloned by ligation-mediated PCR and sequenced on a Genome Analyzer II. Between 700–6800 unique integration events in individual fish were mapped to the zebrafish genome. The data show that introduction of transposase by transgene expression or RNA injection results in an even distribution of transposon re-integration events across the zebrafish genome. SB11 mRNA injection resulted in neoplasms in 10% of adult fish at ∼10 months of age. *T2/OncZ*-induced zebrafish tumors contain many mutated genes in common with human and mouse cancer genes. These analyses validate our mutagenesis approach and provide additional support for the involvement of these genes in human cancers. The zebrafish *T2/OncZ* cancer model will be useful for identifying novel and conserved genetic drivers of human cancers.

## Introduction

In sporadic human cancers the initiating event is proposed to be an oncogenic mutation that activates cell growth pathways and leads to cellular transformation. Subsequent cancer progression and metastasis are associated with genomic instability and defective DNA repair, leading to the accumulation of large numbers of somatic mutations [1], [2], [3]. Large scale sequencing of human cancer genomes has revealed a vast array of somatic mutations in breast, colon, lung, glioma, ovarian, pancreatic, and prostate cancers [4], [5], [6], [7], [8], [9]. A subset of these somatic mutations, termed drivers, are thought to provide a selective growth advantage in cancer cells, promoting cellular transformation and progression to metastasis. The majority of mutations, termed passengers, are carried along as the cancer cell population expands. Distinguishing driver and passenger mutations within the human cancer genome has been a major hurdle in identifying causal mutations in different cancers. A major goal in the field is to correlate cancer genotype with clinically relevant phenotypes in order to develop effective therapies for specific cancers.

One approach to identify new cancer genes and associated causal mutations is to compare the mutation profiles between human cancers and animal cancer models. A successful strategy for cancer gene identification has been developed in mice using the DNA transposon system *Sleeping Beauty* (*SB*) for insertional somatic mutagenesis [10], [11], [12], [13]. The *SB* system was originally reconstituted from inactive elements present in the genomes of salmonid species [14]. Because *SB* shows minimal site specificity aside from the presence of a TA dinucleotide at the integration site [15], it is an effective tool for random mutagenesis. In the mouse somatic mutagenesis cancer models the mutagenic *SB* transposon, *T2/Onc*, was designed to create loss- and gain-of-function mutations similar to those that drive tumorigenesis in sporadic human cancers. The system was shown to induce primarily hematopoietic tumors in adult mice and solid tumors at a lower frequency [10], [11]. Recent modifications to the system using an alternative *T2/Onc* transposon and inducible, tissue-specific transposase sources have been shown to produce a wide range of epithelial-derived carcinomas [16] and tissue-specific cancers including hepatocellular carcinoma [17] and gastrointestinal neoplasms [18]. Analyses of common integration sites across multiple tumor samples have identified putative cancer genes associated with the various types of neoplasms in the mouse *T2/Onc*-induced cancers. Comparative studies with the genes identified in mice and human cancer genomes have revealed a set of mutated genes in common with human cancers [17], [18], [19]. These studies underscore the importance of model systems for validating known cancer genes and identifying novel genes and signaling pathways mutated in sporadic human carcinogenesis.

The development of cancer models in the more distantly related vertebrate species, zebrafish, has demonstrated the conservation of gene pathways underlying cancer [20], [21], [22], [23]. Zebrafish models of human cancers have been created in which tissue-specific over-expression of oncogenes causes soft tissue cancers and solid tumors [24], [25], [26], [27], [28], [29]. Loss of function tumor suppressor lines have been identified that result in solid tumors at varying frequencies [30], [31], [32]. Application of the mouse *SB*-induced mutagenesis strategy [10], [11] would provide a new method to genetically screen for novel cancer genes in zebrafish. Given the high fecundity and inexpensive cost of zebrafish compared to mammalian model systems, large-scale screens approaching saturation mutagenesis of the cancer genome are a possibility in fish. Here we report the adaptation of the *SB T2/Onc* system for somatic mutagenesis in zebrafish. The *T2/Onc* transposon was modified by addition of the carp ß-actin promoter[33], which is widely expressed in zebrafish tissues. We isolated stable transgenic lines that have large copy number concatemers of *T2/OncZ* transposons. Introduction of a transient or constitutive source of *SB11* transposase activates *T2/OncZ* excision from the concatemer and genome-wide re-integration in somatic tissues. Somatic mutagenesis leads to solid tumor induction in ∼10% of adult fish. Comparative analysis of the *T2/OncZ* integration sites from the zebrafish tumor samples reveals a many genes in common with human and mouse cancer genes. This study demonstrates the capability of the *Sleeping Beauty T2/OncZ* system for genome-wide insertional mutagenesis in somatic tissues in zebrafish and its potential for identifying novel cancer genes.

## Results

### A modified *T2/Onc* transposon system for somatic mutagenesis in zebrafish

**Figure 1:**
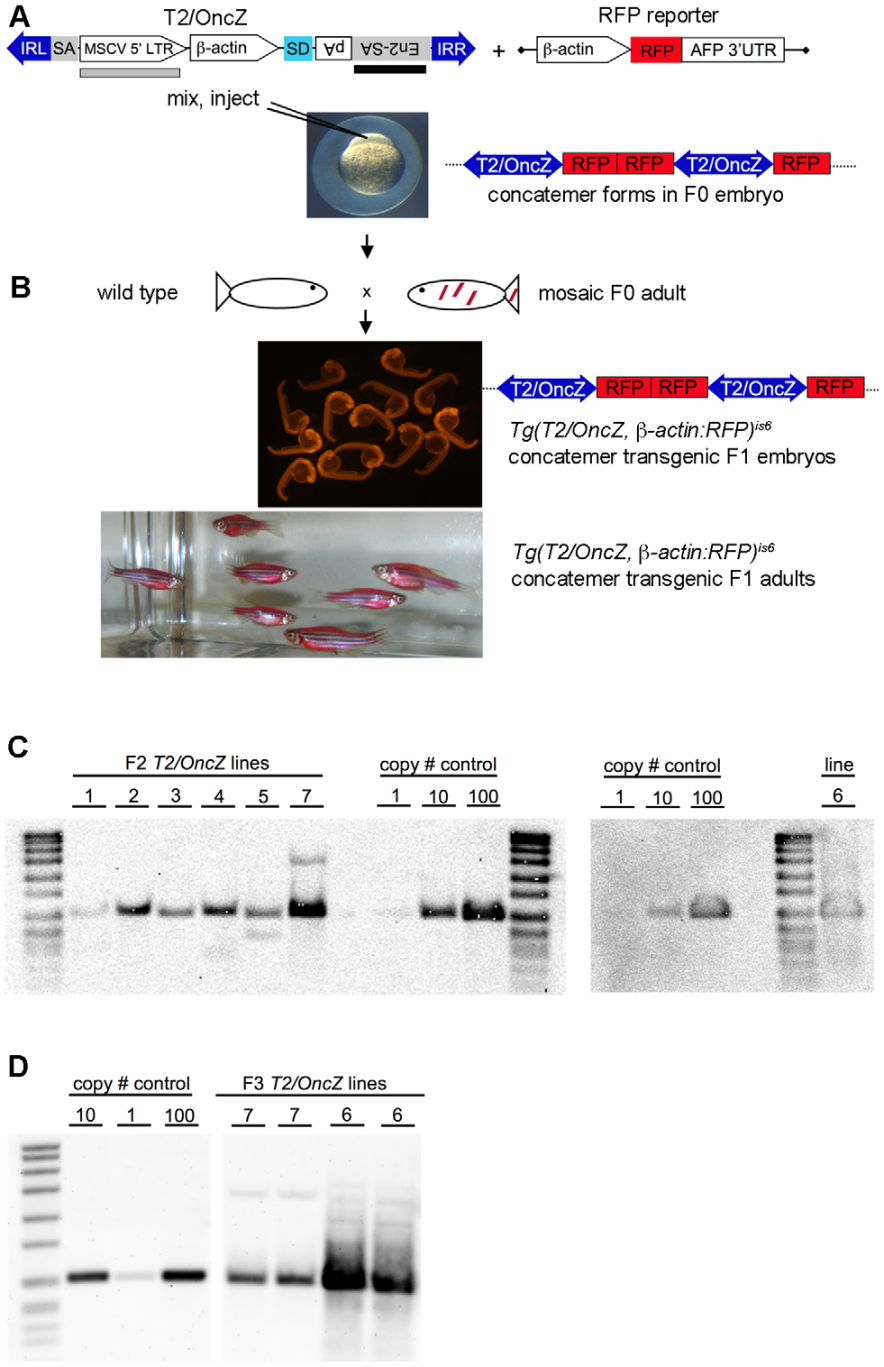
Isolation of transgenic *T2/OncZ* concatemer lines. (*A*) *T2/OncZ* transposon vector and RFP reporter used to isolate concatemers. IRL and IRR, left and right transposon inverted repeats; SA, splice acceptor from intron 1 of ß*-actin* gene; MSCV 5′ LTR, murine stem cell virus 5′ long terminal repeat; ß*-actin*, ß*-actin* promoter minus splice acceptor at 3′ end; SD, splice donor; En2-SA, splice acceptor from mouse *engrailed 2* gene; pA, SV40 polyadenylation sequence; AFP, ocean pout *antifreeze protein* 3′ UTR; black bar represents probe used on genomic Southerns shown in panel *C*. Grey box represents probe used on genomic Southerns shown in panel *D*. Linear DNA fragments of *T2/OncZ* and the ß*-actin:RFP* reporter gene were mixed and co-injected into 1-cell zebrafish embryos. (*B*) Adult F0 founders were outcrossed to wild type and transgenic F1 embryos identified by ubiquitious RFP fluorescence. (*C*) Genomic Southern blots to estimate transposon copy number in *Tg*(*T2/OncZ*, ß*-actin:RFP*) concatemer lines 1–7. DNA was isolated from F2 generation heterozygous adults. (*D*) Genomic Southern blot of DNA isolated from F3 generation heterozygous *Tg*(*T2/OncZ*, ß*-actin:RFP*)*^is7^* and *Tg*(*T2/OncZ*, ß*-actin:RFP*)*^is6^* adults. Plasmid *pT2/OncZ* was loaded as reference in copy # control lanes.

The *T2/Onc* transposon used in previous mouse models contains elements to terminate transcription and promote over-expression after the transposon inserts in or near a gene [10], [11]. The MSCV 5′ LTR promoter in *T2/Onc* drives high-level expression in hematapoietic tissues in mouse. However, it was unknown how well this promoter would be expressed when integrated into the zebrafish genome. In addition, we wanted to build a *T2/Onc* transposon that contains a promoter that is widely expressed in various tissues and cell types. We altered *T2/Onc* by addition of the carp ß-actin promoter [33], which has been used extensively to drive constitutive and ubiquitous expression in fish species [34]. The promoter is defined by 2.5 Kb of upstream sequence, the first exon which is non-coding, and the first intron which contains elements necessary for high level expression from the promoter [35]. The splice acceptor sequences (SA) at the 3′ end of intron 1 were removed to force splicing out of the transposon and into downstream exons after insertion into a gene. The modified ß-actin promoter was cloned into *T2/Onc* behind the MSCV 5′ LTR to create *T2/OncZ* (Fig. 1 *A*).

To isolate transgenic fish carrying large arrays of *T2/OncZ* we used a standard transgenesis method of injecting linear DNA fragments into the 1-cell zebrafish embryo. A 4.9 Kb linear fragment containing *T2/OncZ* was released from the plasmid vector and mixed with a linear fragment containing a widely expressed ß*-actin:RFP* reporter cassette [36] before injection (Fig. 1 *A*). After injection into the 1-cell embryo the *T2/OncZ* and RFP reporter fragments ligate via the non-homologous end-joining pathway to form a concatemer that inserts randomly into the genome. We used the RFP reporter to screen for transgenic progeny and to follow the concatemer through subsequent generations. Out of 54 founder F0 adults screened by outcrossing with wild type zebrafish, we identified 7 F0s that produced from 3–10% RFP+ embryos in the F1 generation (Fig 1 *B*). The 7 independent F1 lines were raised and outcrossed to create an F2 generation. The transgenic embryos are easily distinguished by ubiquitous RFP expression, and adults appear red due to high levels of RFP expression from multiple ß*-actin:RFP* reporter cassettes in the concatemer (Fig. 1 *B*).

The copy number of the *T2/OncZ* transposon in the concatemer lines was determined by genomic Southern blot analysis of DNA isolated from F2 adults (Fig. 1 *C*). Plasmid DNA containing the *T2/OncZ* transposon was loaded in amounts corresponding to 1, 10 or 100 copies/genome and used as a reference to estimate the transposon copy number in each line by densitometry. The number of transposons in the concatemers ranged from ∼5 to >100 copies (Fig. 1 *C*). Additional Southern blot analyses consistently indicated that the number of transposons in the *Tg*(*T2/OncZ*, ß*-actin:RFP*)*^is6^* line 6 far exceeded 100. To clarify this issue, we again examined the estimated copy number of transposons in the line 7 and line 6 concatemers in the F3 generation (Fig. 1 *D*). The results indicate that line *Tg*(*T2/OncZ*, ß*-actin:RFP*)*^is7^* contains 70–100 transposons, while *Tg*(*T2/OncZ*, ß*-actin:RFP*)*^is6^* contains >500 transposons. While our analysis provides only an estimate of the actual number of *T2/OncZ* transposons in the concatemers, the important point, as demonstrated below, is that both concatemer lines have a large number of transposons that are capable of robust transposition and reintegration in somatic tissues. We have maintained lines *Tg*(*T2/OncZ*, ß*-actin:RFP*)*^is7^* and *Tg*(*T2/OncZ*, ß*-actin:RFP*)*^is6^* and observed that the concatemers appear stable, behave like a single copy locus, and show a Mendelian pattern of inheritance through 5 generations to date (data not shown).

**Figure 2:**
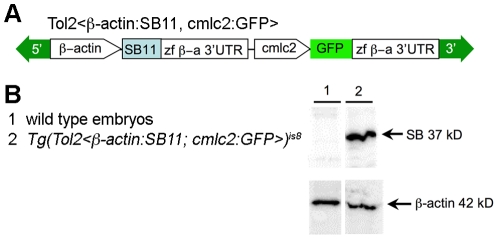
Isolation of transgenic zebrafish expressing constitutive SB11 transposase. (*A*) Diagram of *miniTol2* vector containing a constitutive ß*-actin* promoter: SB11 cDNA cassette. 5′ and 3′, Tol2 inverted terminal repeats; zf ß-a 3′UTR, zebrafish ß*-actin* 3′ UTR; *cmlc2*, zebrafish *cardiac myosin light chain 2* promoter. (*B*) Western blot demonstrates the expression of SB11 in *Tg*(*Tol2<*ß*-actin:SB11*, *cmlc2:GFP>*) transgenic embryos. The blot was stripped and re-probed with an anti-ß*-actin* antibody for loading control.

To isolate a transgenic line that constitutively expresses SB11 transposase [37], a transgenesis vector was assembled inside the mini*Tol2* transposon [38] for *Tol2* transposase-mediated integration into the genome. The carp ß-actin promoter [33] was used to express *SB11* cDNA from within the transposon (Fig. 2 *A*). For a marker of transgenesis, a heart-specific reporter cassette containing the cardiac myosin light chain 2 (*cmlc2*) promoter [39], [40] driving EGFP was cloned into the mini*Tol2* vector 3′ to the *SB11* expression cassette. To isolate transgenic lines carrying single copies of the ß-*actin:SB11, cmlc2:EGFP* transposon, the vector and *Tol2* mRNA were co-injected into 1-cell embryos. Founder F0 embryos were raised to adulthood and screened for transgenic progeny by outcrossing with wild type zebrafish and scoring EGFP expression in the heart. Nine independent lines were isolated that carry a single *Tol2*<ß*-actin:SB11*, *cmlc2:GFP>* transgene insertion and two were maintained (Table 1). Expression of the SB11 transposase was confirmed in line *Tg*(*Tol2<*ß*-actin:SB11*, *cmlc2:GFP>*)*^is8^* by western blotting of 5-hour post fertilization (hpf) embryos (Fig. 2 *B*).

**Table 1:** Transgenic zebrafish lines isolated in this study.

Transgenic line	Transgene copy number	Notes
*Tg*(*T2/OncZ*,ß*-actin:RFP*)*^is7^*	∼70 copy concatemer	RFP reporter integrated into concatemer
*Tg*(*T2/OncZ*,ß*-actin:RFP*)*^is6^*	∼500 copy concatemer	RFP reporter integrated into concatemer
*Tg*(*Tol2<*ß*-actin:SB11*, *cmlc2:GFP>*)*^is8^*	single *Tol2* transposon with SB11 transposase cassette	contains heart-specific GFP reporter cassette
*Tg*(*Tol2<*ß*-actin:SB11*, *cmlc2:GFP>*)*^is9^*	single *Tol2* transposon with SB11 transposase cassette	contains heart-specific GFP reporter cassette

**Figure 3:**
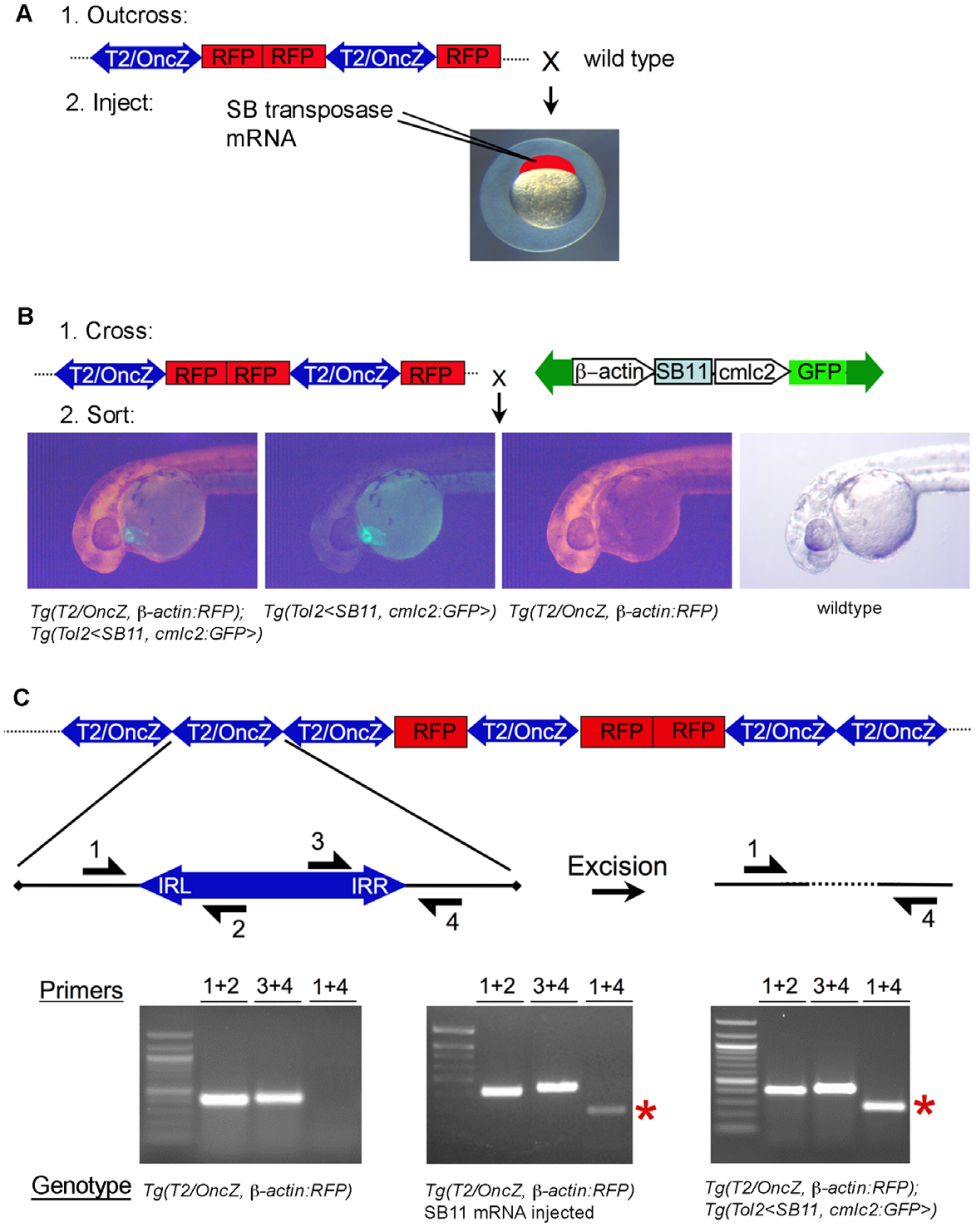
Two strategies for *T2/OncZ* insertional mutagenesis in zebrafish somatic tissues. (*A*) Methods 1: Injection of *in vitro* transcribed SB11 mRNA at the 1-cell stage. (*B*) Method 2: Genetic cross between *Tg*(*T2/OncZ*, ß*-actin:RFP*) (*T2/OncZ*) and constitutive *Tg*(*Tol2<*ß*-actin:SB11*, *cmlc2:GFP>*) (ß*-actin:SB11*) fish. At 24 hpf embryos are sorted into 4 progeny classes. (*C*) Excision PCR assay on 5 dpf larvae to demonstrate mobilization of transposons out of the concatemer in the presence of SB11 transposase. Primers 1 and 4 amplify a 220 bp band (red asterisk) flanking the transposon excision site in the concatemer. Left panel, *Tg*(*T2/OncZ*, ß*-actin:RFP*)*^is6^* larvae; middle panel, SB11 injected *Tg*(*T2/OncZ*, ß*-actin:RFP*)*^is6^* larvae; right panel, double transgenic *Tg*(*T2/OncZ*, ß*-actin:RFP*)*^is6^*; *Tg*(*Tol2<*ß*-actin:SB11*, *cmlc2:GFP>*) larvae.

To demonstrate the activity of the SB11 transposase and the ability of the *T2/OncZ* transposon to excise from the concatemer, we used a PCR-based assay as described previously [10]. We used two methods for introducing SB11 transposase into the *Tg*(*T2/OncZ*, ß*-actin:RFP*) transgenic fish. In the first method, *in vitro* transcribed SB11 mRNA was injected into 1-cell embryos from *Tg*(*T2/OncZ*, ß*-actin:RFP*) fish (Fig. 3 *A*). In the second method, double transgenic embryos were recovered by crossing the *Tg*(*T2/OncZ*, ß*-actin:RFP*) line to the *Tg*(*Tol2<*ß*-actin:SB11*, *cmlc2:GFP>*)*^is8^* line that expresses SB11 under the control of the ß*-actin* promoter (Fig. 3 *B*). Embryos were aged to 5 day post fertilization (dpf) and genomic DNA isolated from the larvae for PCR. In the *T2/OncZ* concatemer each transposon has ∼200 bp of plasmid vector sequence flanking the transposon on both sides (Fig. 3 *C*). Primers 1 and 4 are complementary to these flanking sequences and are expected to amplify an ∼200 bp product only if the transposon has excised from the concatemer and the excision site is repaired (Fig. 3 *C*). The excision PCR product, which indicates *T2/OncZ* transposons have excised from the concatemer, is present in embryos doubly heterozygous for the *T2/OncZ* concatemer and *Tg*(*Tol2<*ß*-actin:SB11*, *cmlc2:GFP>*)*^is8^* transgenes (Fig. 3 *C*, asterisk), but absent from embryos carrying the concatemer alone. A similar result was obtained when transposase was supplied by injection of SB11 mRNA into *Tg*(*T2/OncZ*, ß*-actin:RFP*)*^is6^* embryos (Fig. 3 *A*, *C*, *asterisk*). We confirmed that the PCR products represent the excision footprint by sequencing (data not shown). These results demonstrate that the *T2/OncZ* concatemers contain functional transposons that can be excised from the concatemer. In addition, the results show that the SB11 transposase expressed from either an mRNA or a transgene can actively promote mobilization of transposons out of the concatemer.

### Demonstration of genome-wide *T2/OncZ* transposon integration after transient or constitutive exposure to *SB11* transposase

To test whether the *T2/OncZ* system is effective at producing genome-wide somatic insertional mutations, we examined the extent of re-integration following injection of *SB11* mRNA into 1-cell stage embryos that had either the *Tg*(*T2/OncZ*, ß*-actin:RFP*)*^is6^* (∼500 copies) or *Tg*(*T2/OncZ*, ß*-actin:RFP*)*^is7^* (∼70 copies) concatemer (Fig. 3 *A*, Table 2, Experiments 1 and 2). At one year of age, three fish (1a, 1b, and 6) that had developed large tumors (described below) were sacrificed and DNA was isolated separately from muscle tissue (C, control) and tumor tissue (T, tumor). Junction fragments from transposon integration sites were amplified using a ligation-mediated PCR protocol [16] with barcoded primers adapted for next generation sequencing on the Illumina/Solexa platform (Table 3). The sequences were trimmed and the transposon–genomic DNA junction fragments were mapped to the zebrafish v8 RefSeq genome (http://www.sanger.ac.uk/resources/zebrafish). We recovered 1630,717, and 2479 unique integration sites in control 1aC, 1bC, and 6C samples, respectively (Table 4 and Dataset S1), that mapped to TA dinucleotides in the zebrafish genome. These data indicate that the short burst of transposase expression from mRNA injection in the embryo was enough to trigger widespread transposon mobilization and re-integration.

**Table 2:** Somatic mutagenesis experiments performed in this study.

				Mutant Class			Control Siblings		
Experi-ment	Parental Genotype	Parental Genotype	Transposase Source	Genotype	Tumor Incidence	*tp53^M214K^* Genotype	Genotype	Tumor Incidence	*tp53^M214K^* Genotype
1	*Tg(T2/OncZ)^is6^/+*	*Tg(T2/OncZ)^is6^/+*	*SB11 mRNA injection*	*T2/OncZ*/+	3/29*	NA	+/+	0/20	NA
2	*Tg(T2/OncZ)^is7^/+* ; *tp53*/+	*+/+* ; *tp53*/*tp53*	*SB11 mRNA injection*	*T2/OncZ*/+	1/48#	*tp53*/+	+/+	0/55	NA
3	*Tg(T2/OncZ)^is6^/+*	*Tg*(ß*-a:SB11*)*^is8^/+*	constitutive transgene	*T2/OncZ*/+ ; ß*-a:SB11*/+	0/40	NA	*T2/OncZ*/+ ; +/+	0/41	NA
4	*Tg(T2/OncZ)^is6^/+* ; *tp53*/+	*Tg*(ß*-a:SB11*)*^is8^/+* ; *+/+*	constitutive transgene	*T2/OncZ*/+ ; ß-*a:SB11*/+	2/3∧	*tp53*/+	*T2/OncZ*/+ ; +/+	0/18	NA

Tumor incidence measured at 1–1.5 years.

*tp53^M214K^* allele previously described in Berghmans et al., 2005.

*Tumor 1aT, intestinal mass, pathology not determined; tumor 1bT, spindle cell sarcoma; tumor 1cT, abdominal mass, pathology not determined.

# Tumor 6T, carcinoma.

∧ Tumor 2T, mixed spindle cell and histiocytic sarcoma; tumor 8T, spindle cell sarcoma.

**Table 3:** 5′-3′ Sequence of barcoded oligos for ligation-mediated PCR of *T2/OncZ* transposon-genomic DNA junction fragments.

Primer	Illumina Adaptor Sequence	Barcode	*T2* transposon arm sequence
BC1	AATGATACGGCGACCACCGAGATCTACACTCTTTCCCTACACGACGCTCTTCCGATCT	AGGAGT	TGTATGTAAACTTCCGACTTCAACTG
BC2	AATGATACGGCGACCACCGAGATCTACACTCTTTCCCTACACGACGCTCTTCCGATCT	GCGAGT	TGTATGTAAACTTCCGACTTCAACTG
BC3	AATGATACGGCGACCACCGAGATCTACACTCTTTCCCTACACGACGCTCTTCCGATCT	CTGAGT	TGTATGTAAACTTCCGACTTCAACTG
BC4	AATGATACGGCGACCACCGAGATCTACACTCTTTCCCTACACGACGCTCTTCCGATCT	AACAGT	TGTATGTAAACTTCCGACTTCAACTG
BC5	AATGATACGGCGACCACCGAGATCTACACTCTTTCCCTACACGACGCTCTTCCGATCT	GGCAGT	TGTATGTAAACTTCCGACTTCAACTG
BC6	AATGATACGGCGACCACCGAGATCTACACTCTTTCCCTACACGACGCTCTTCCGATCT	TCCAGT	TGTATGTAAACTTCCGACTTCAACTG
BC7	AATGATACGGCGACCACCGAGATCTACACTCTTTCCCTACACGACGCTCTTCCGATCT	GATAGT	TGTATGTAAACTTCCGACTTCAACTG
BC8	AATGATACGGCGACCACCGAGATCTACACTCTTTCCCTACACGACGCTCTTCCGATCT	CGTAGT	TGTATGTAAACTTCCGACTTCAACTG
BC9	AATGATACGGCGACCACCGAGATCTACACTCTTTCCCTACACGACGCTCTTCCGATCT	ACTAGT	TGTATGTAAACTTCCGACTTCAACTG
BC10	AATGATACGGCGACCACCGAGATCTACACTCTTTCCCTACACGACGCTCTTCCGATCT	GAAGGT	TGTATGTAAACTTCCGACTTCAACTG
BC11	AATGATACGGCGACCACCGAGATCTACACTCTTTCCCTACACGACGCTCTTCCGATCT	AGAGGT	TGTATGTAAACTTCCGACTTCAACTG
BC12	AATGATACGGCGACCACCGAGATCTACACTCTTTCCCTACACGACGCTCTTCCGATCT	CCAGGT	TGTATGTAAACTTCCGACTTCAACTG

**Table 4:** Analysis of *T2/OncZ* integration events in control tissue and tumor tissue after exposure to transient or constitutive SB11 transposase.

Sample	*T2/OncZ* concatemer line	Transposase Source	Integrations/Genome	Integrations in Genic Regions*	# genes with ≥1 integration∧	# genes with >2 integrations	# genes with >3 integrations	# genes with >4 integrations	% genes with multiple integrations
1aC	∼500 copy	SB11 mRNA	1630	1340	1169	135	19	3	11.5%
1bC	∼500 copy	SB11 mRNA	717	589	491	54	16	5	11%
6C	∼70 copy	SB11 mRNA	2479	2035	1626	269	71	18	16.5%
1aT	∼500 copy	SB11 mRNA	2596	2143	1679	302	81	28	18%
1bT	∼500 copy	SB11 mRNA	1831	1497	1220	194	44	11	15.9%
6T	∼70 copy	SB11 mRNA	1818	1480	1256	163	29	11	13%
1C	∼500 copy	*Tg*(ß*-a:SB11*)	4446	3567	2769	571	142	32	20.6%
2C	∼500 copy	*Tg*(ß*-a:SB11*)	5363	4280	3232	762	182	56	23.6%
3C	∼500 copy	*Tg*(ß*-a:SB11*)	6842	5469	3849	1092	333	102	28.4%
5C	∼500 copy	*Tg*(ß*-a:SB11*)	4470	3666	2850	599	142	22	21%
2T	∼500 copy	*Tg*(ß*-a:SB11*)	2306	1848	1598	214	21	5	13.4%
8T	∼500 copy	*Tg*(ß*-a:SB11*)	2307	1868	1599	213	40	11	13.3%

C = control muscle tissue from tumor born fish (1a, 1b, 6) or from tumor free control fish (1C, 2C, 3C, 5C).

T = tumor tissue from fish 1a, 1b, 6, 2 and 8.

*Integrations in Genic Region is defined as an integration in the 5′, 3′, intronic or exonic region of a gene.

∧ The number of individual genes with one or more independent integration events.

**Figure 4:**
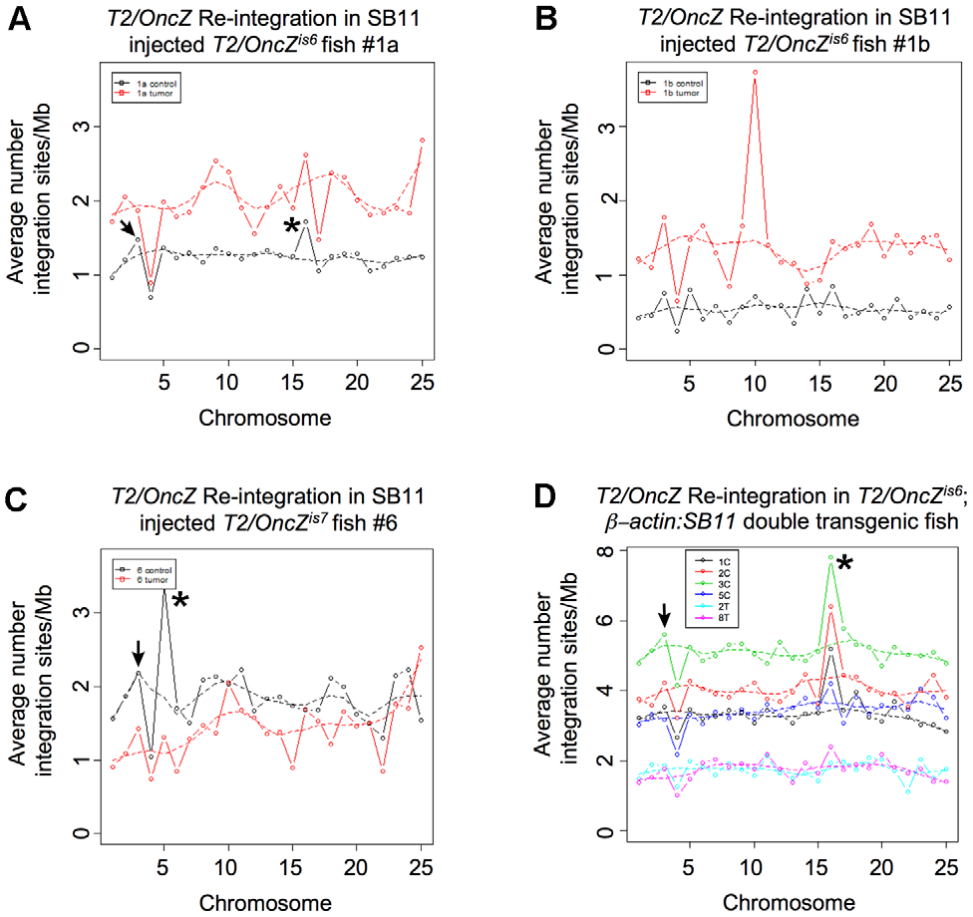
Re-integration of *T2/OncZ* after transient or constitutive SB11 transposase expression. (*A*, *B*, *C*) The average number of re-integration sites per Mb plotted across all 25 chromosomes for tumor (T) and control (C) muscle samples from fish #1a, #1b and #6. Transposase source was supplied as *SB11* mRNA injected at the 1-cell stage into *Tg*(*T2/OncZ*, ß*-actin:RFP*)*^is6^* (fish #1a, #1b) or *Tg*(*T2/OncZ*, ß*-actin:RFP*)*^is7^* (fish #6) embryos. (*D*) The average number of re-integration sites per Mb plotted across all 25 chromosomes for control samples from fish 1C, 2C, 3C, and 5C, and tumor samples from fish 2T and 8T. Fish were double heterozygous *Tg*(*T2/OncZ*, ß*-actin:RFP*)*^is6^* ;*Tg*(*Tol2<*ß*-actin:SB11*, *cmlc2:GFP>*).

The *T2/OncZ* re-integration site data for individual samples was plotted and revealed that in each sample the integration events mapped to every chromosome (Fig. 4 *A–C*, black line plots). The average number of integration sites per Mb across each chromosome was ∼0.5, 1 (samples 1bC and 1aC), or 2 (sample 6C) and appeared evenly distributed across the genome with two exceptions. First, in each sample a large number of re-integration sites mapped to chromosome 3 (Figure 4, *A–C*, arrows), where the zebrafish ß*-actin* gene is located. These are most likely re-integration events in the ß*-actin* promoter present in the *T2/OncZ* transposon (Dataset S1). Second, in each sample one chromosome has a higher frequency of re-integration events than the rest (Figure 4 *A–C*, asterisks). For example, in *Tg*(*T2/OncZ*, ß*-actin:RFP*)*^is6^* sample 1a the peak on chromosome 16 indicates there are ∼1.5 times as many events as on the other chromosomes (Fig. 4 *A* asterisk). In *Tg*(*T2/OncZ*, ß*-actin:RFP*)*^is7^* sample 6C, the highest frequency of re-integration events mapped to chromosome 5 (Fig. 4 *C* asterisk). The cluster of integration sites most likely represents transposon excision and re-integration to locations linked to the concatemer, a previously reported phenomenon [10], [11] that is discussed further below. Interestingly, in control tissue from fish 1b the distribution of integration sites was relatively even across each chromosome, without an obvious peak representing linkage to the predicted concatemer location on chromosome 16 (Fig. 4 *B*). However, analysis of the integration sites from the tumor tissue isolated from fish 1b shows a peak on chromosome 10 (Fig. 4 *B* asterisk). One explanation for this discrepancy is that a genomic rearrangement or translocation of part of the concatemer occurred in the developing embryo after injection of SB11 transposase mRNA. Subsequently, the cells that inherited the rearrangement went on to contribute to the tumor in the adult. This is a reasonable explanation, given that transposition from a concatemer has been previously shown in mice to cause genomic rearrangements at the concatemer chromosomal site [41].

The experiments described above demonstrated that supplying a transient source of SB11 transposase by injection of mRNA into the embryo results in a robust level of transposition. We next compared the extent of transposon mobilization after transient transposase expression with the constitutive SB11 transgene. To do this we cloned and analyzed the distribution of transposon integration sites in the genomes of four three-month-old fish that were doubly transgenic for the *Tg*(*T2/OncZ*, ß*-actin:RFP*)*^is6^* concatemer and the *Tg*(*Tol2<*ß*-actin:SB11*, *cmlc2:GFP>*)*^is8^* transgene (Figure 3 *B*, Table 4 Experiment 3). Between 4446–6842 unique integration sites were identified per tissue sample (Table 4 and Dataset S1). The average number of integration sites per Mb was between 2 and 6 in samples 1C, 2C, 3C and 5C (Fig. 4 *D*), at least twice the number seen after supplying SB11 transposase transiently in the embryo (Fig. 4 *A–C*). This could reflect the continued transposition driven by the constitutive transposase through adult stages.

In each of the four *Tg*(*T2/OncZ*, ß*-actin:RFP*)*^is6^*; *Tg*(*Tol2<*ß*-actin:SB11*, *cmlc2:GFP>*)*^is8^* samples a peak of integration was observed on chromosome 16 (Fig. 4 *D*). This was consistent with what was observed in SB11 injected fish 1a (Fig. 4 *A*) and provides additional support for the location of *Tg*(*T2/OncZ*, ß*-actin:RFP*)*^is6^* on chromosome 16. Assuming that transposons should re-integrate randomly across the genome, we calculated the expected frequency of integration events for each chromosome based on what proportion of the genome each chromosome represents in Mb. Chi-square tests of the observed and expected frequencies of integration events in a double transgenic *Tg*(*T2/OncZ*, ß*-actin:RFP*)*^is6^* ; *Tg*(*Tol2<*ß*-actin:SB11*, *cmlc2:GFP>*)*^is8^*fish (sample 5C) indicate the distribution of transposon integrations sites was significantly different than expected (two tailed *P*<0.0001). Although the expected number of integration events is an approximation based on size and not the distribution of TA dinucleotides on each chromosome, the data are consistent with linkage of the *Tg*(*T2/OncZ*, ß*-actin:RFP*)*^is6^* concatemer to chromosome 16. Similarly, chi-square tests of integration sites in the ∼70 copy number concatemer sample *Tg*(*T2/OncZ*, ß*-actin:RFP*)*^is7^* 6C (Fig. 4 *C*) indicated a significant difference in the distribution compared to the expected (two tailed *P*<0.0001). The analysis provides additional evidence that the *Tg*(*T2/OncZ*, ß*-actin:RFP*)*^is7^* concatemer is linked to chromosome 5.

Within each sample from double transgenic ß*-actin:SB11^is8^* ; *T2/OncZ^is6^* fish the number of integration events that are in or near a gene ranged from 3567–5469, or 80–82% of the total integration events that mapped to the genome (Table 4). Further analysis revealed that in each double transgenic sample 22–28% of the genes have multiple independent insertion sites (Table 4). The number of independent integrations in a gene ranged from 1 to >4 in each sample, with 22, 32, 56 and 102 genes in the different samples having 4 or more independent integration events (Table 4, samples 5C, 1C, 2C, 3C). Surprisingly, multiple independent insertions were also detected in 11–16% of the genes after transient expression of transposase by SB11 mRNA injection into the embryo (Table 4). Multiple independent events in a gene in one tissue sample could represent intragenic excision and re-integration, as described previously in mouse [10], [11], [17], [18]. The ability to detect these multiple events may result from the massive sequencing capability of the Illumina/Solexa platform, compared with previous high throughput methods. In summary, both methods we tested for introducing transposase into the *T2/OncZ* concatemer lines resulted in transposition and genome-wide re-integration.

### *T2/OncZ* mutagenesis leads to tumor induction after transient or constitutive exposure to *SB11* transposase

**Figure 5:**
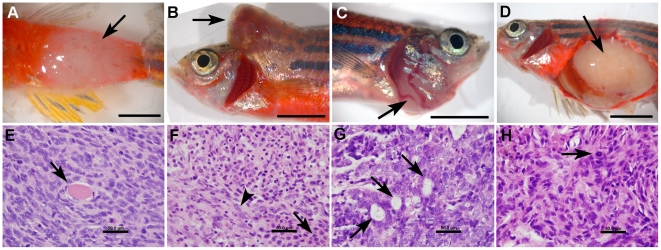
Histopathologic features of solid tumors in *T2/OncZ* mutagenized fish. (*A–D*) gross images of neoplasms in fish 1a, 2, 6, and 8 respectively. (*E–H*) Histopathology of zebrafish neoplasms: Hematoxylin and Eosin stained sections at 1000× magnification. (*E*) Spindle cell sarcoma from fish 1a, note entrapped skeletal muscle fibers (arrow). (*F*) Mixed neoplasm from fish 2, neoplastic round cells (arrow) were intermixed with neoplastic spindle shaped cells (arrow head). (*G*) Carcinoma from fish 6, neoplastic cells were arranged into multiple acinar structures (arrow). (*H*) Spindle cell sarcoma from fish 8, note the mitotic figure (arrow). Scale bar A–D, 0.5 cm; E–H, 50 um.

To determine if transposon re-integration in the zebrafish *T2/OncZ* system would lead to tumorigenesis, we monitored the *Tg*(*T2/OncZ*, ß*-actin:RFP*)*^is6^* zebrafish exposed to transient or constitutive transposase for gross evidence of neoplasms or tumors. In a wild type background, 10% (3/29) of the >500-copy number concatemer *Tg*(*T2/OncZ*, ß*-actin:RFP*)*^is6^* adults that had been injected with *SB11* mRNA at the 1-cell stage developed gross tumors beginning at about 1 year of age (Table 2, Experiment 1). Twenty control siblings that were injected and reared alongside the *Tg*(*T2/OncZ*, ß*-actin:RFP*)*^is6^* positive fish showed no evidence of neoplasms. Naturally occurring neoplasms have been reported in zebrafish with varying frequencies [42], [43], [44], however, we have not observed spontaneous tumors in the *T2/OncZ* transgenic lines or in the wild type populations of fish raised in our facility. We harvested the tumor from one of the mutagenized fish (1a) and performed both histopathology and transposon re-integration site analysis. In fish 1a a mesenchymal neoplasm was located in the abdominal body wall (Fig. 5 *A*). The neoplasm consisted of densely packed and highly cellular cords of spindle shaped cells within a fine fibrous stroma; a microscopic appearance consistent with spindle cell sarcoma (Fig. 5 *E*). Neoplastic cells invaded extensively into and separated the skeletal muscle of the abdominal wall. Together, these results indicate that *SB* transposon mutagenesis induces tumors in zebrafish adults.

We examined whether predisposing fish to cancer in a tumor suppressor mutant background would accelerate tumor onset and increase the frequency of tumors after *T2/OncZ* mutagenesis. Homozygous *tp53^M214K^* mutant zebrafish are susceptible to development of peripheral nerve sheath tumors [31]. *SB11* mRNA was injected into embryos from a cross between the 70 copy number *Tg*(*T2/OncZ*, ß*-actin:RFP*)*^is7^* concatemer line and homozygous *tp53^M214K^* zebrafish, so that all progeny were heterozygous for the *tp53^M214K^* allele (Table 2, Experiment 2). Transgenic and non-transgenic progeny were monitored for evidence of neoplasms (Table 2). At 9 months of age, one adult zebrafish (1/48–2%) that was injected with *SB11* mRNA developed a large neoplasm rostral and ventral to the pectoral fin (Fig. 5 *C*). None of the 55 non-transgenic siblings developed tumors. The neoplasm in the fish was composed of densely packed nests of cuboidal to polygonal cells that had moderate anisocytosis and anisokaryosis. In multiple foci neoplastic cells were arranged into tubular/acinar structures (Fig. 5 *G*). The morphology of neoplastic cells suggests an epithelial origin, indicating a carcinoma, which is different from the typical MPNSTs that arise due to *p53* heterozygosity. The *tp53* genotype of DNA isolated from the tumor and control muscle tissue of the fish was determined by amplification and sequencing of a 200 bp fragment surrounding the *M214K* lesion. The results indicate that the control muscle tissue and tumor tissue were heterozygous for the *tp53^M214K^* allele (data not shown). It is possible that DNA from stromal tissue in the tumor contributed to the presence of the wild type allele in the genotyping data. However, given the absence of tumor induction in 55 non-transgenic control siblings, it is less likely that the tumor appeared after spontaneous loss of the wild type *tp53^M214K^* allele. The lower frequency of tumor induction observed in these experiments (2%), compared to the frequency for the high copy number concatemer (10%), suggests transposon copy number positively correlates with tumor induction frequency. Predisposing fish with a single copy of mutant *tp53* likely did not compensate for the lower transposon dosage in the lower copy number concatemer line.

The introduction of transposase by mRNA injection was able to induce tumors in wild type and *p53* heterozygous adult *T2/OncZ* fish, albeit at a low frequency. We expected that a continuous, transgenic source of SB11 transposase would result in an increase in tumor frequency and a decrease in latency, particularly in the predisposed *p53* background. However, zero out of 40 double transgenic *T2/OncZ^is6^* ; ß*-actin:SB11^is8^* adults developed tumors by 1 year of age (Table 2, Experiment 3). Additionally, in crosses between the constitutive ß*-actin:SB11* transposase and the ∼500 copy number *T2/OncZ* concatemer line that was heterozygous for the *tp53^M214K^* allele, only three out of approximately 20 double transgenic progeny survived (Table 2, Experiment 4). However, at 1.5 years two of the three adults developed gross neoplasms (Fig. 5 *B*, *D*), while 0/18 siblings that did not inherit the ß*-actin:SB11* transgene showed signs of tumors (Table 2, Experiment 4). These data are consistent with the idea that mutant *p53* predisposes fish to increased tumorigenesis and further supports the observation that transposon dosage in the concatemer correlates with tumor incidence. In fish 2 we identified a neoplasm in the dorsal cervical region (Fig. 5 *B*). This neoplasm appeared to be composed of two neoplastic cell populations each with distinct morphology. The first cell type was characterized by loosely packed sheets of round cells within a fine fibrous stroma (Fig. 5 *F*). The second neoplastic population consisted of densely packed and highly cellular spindle shaped cells within a fibrous stroma (Fig. 5 *F*). Neoplastic cells in both populations had had moderate anisocytosis and anisokaryosis. Our interpretation of these findings was that this was a mixed neoplasm containing two neoplastic cell phenotypes, mesenchymal and round cell. These morphologies are consistent with spindle cell sarcoma and histiocytic sarcoma, respectively. In fish 8 there was a mesenchymal neoplasm located in the abdominal cavity with abundant fibrous tissue that may indicate further differentiation and classification as fibrosarcoma. Neoplastic cells had moderate anisocytosis and anisokaryosis (Fig. 5 *H*). The morphology of this neoplasm is consistent with spindle cell sarcoma. We determined the *tp53* genotype of control muscle tissue and tumor tissue from both fish 2 and fish 8 using PCR and sequencing. Like control tissue, the tumor DNA remained heterozygous for the *tp53^M214K^* allele. These results are consistent with *T2/OncZ* mutagenesis driving tumorigenesis in these neoplasms, as opposed to loss of heterozygocity at the *tp53* locus.

### *T2/OncZ* transposon integration site analysis in neoplastic tissue

We examined the profile of transposon sites from neoplastic tissues (Table 4, Dataset S1) and compared the gene insertion list with human and mouse cancer genome databases. We isolated DNA from tumor tissues 1aT, 1bT, 6T, 2T and 8T. A list of all integration sites for every tumor and control tissue is provided in Dataset S1. The integration site list was filtered according to a method previously used in the analysis of *T2/Onc* integration sites from mouse [16]. This analysis is designed to create a conservative estimate of the genes that contribute to cellular transformation based on their representation in the Illumina sequence data set. Using this method we identified in each tissue sample the integration sites two standard deviations above the mean, which potentially represent transposon integrations that were clonally expanded during tumor growth (Table 5, Dataset S2). The number of annotated genes for all zebrafish tumor samples (T) after filtering is 149. Interestingly, insertions at different locations in the GALNTL6 gene that encodes N-acetylgalactosaminyltransferase-like 6 were observed in two tumor samples, 2T and 8T.

**Table 5:** Number of *T2/OncZ* re-integration sites/sample after filtering.

Sample	# of sites	# of mapped reads	Avg. read #	standard deviation	Cut off value	# of sites above Cut off value	# annotated genes
1aC	1621	51475	31	388	806	10	7
1bC	693	43805	62	347	758	14	10
6C	2452	182394	74	526	1126	51	45
1aT	2578	239283	93	1061	2215	34	26
1bT	1826	194852	107	2027	4160	5	4
6T	1811	74780	41	529	1099	11	9
1C	4220	345805	78	305	688	155	127
2C	5353	520800	97	316	730	238	193
3C	6834	718548	105	359	824	247	185
5C	4461	215711	48	345	738	70	47
2T	2304	205197	89	355	800	90	72
8T	2308	228567	99	571	1243	55	49

Samples 1aC, 1bC, and 6C are from normal muscle tissue from fish 1a, 1b, 6.

Samples 1aT, 1bT, and 6T are from tumor tissue from fish 1a, 1b, 6.

Samples 1C, 2C, 3C, and 5C are from ß*-actin:SB11;T2/OncZ^is6^* double transgenic control fish.

Samples 2T and 8T are from tumor tissues from ß*-actin:SB11;T2/OncZ^is6^* double transgenic fish.

Reads that mapped to the ß-actin promoter were removed from each sample before calculating values.

Cut off value is equal to two standard deviation units above the average read # in that sample.

# annotated genes is the # of sites within an annotated gene or positioned 5′ or 3′ to an annotated gene.

We then compared the gene list from the zebrafish tumors with the mouse retroviral tagged cancer gene database (RTCGD: retroviral tagged cancer gene database, http://RTCGD.ncifcrf.gov) [45]. Retroviral insertions were present in the mouse homologs of 10 genes tagged in the zebrafish tumor samples: *Brd2*, *Cirbp*, *Fgf8*, *Hexim1*, *Map2k5*, *Mmp14*, *Ncoa2*, *Slc30a5*, *Sox4*, and *Sox5* (Table 6). Additionally, a *T2/OncZ* transposon insertion in *Snapc3* was observed in tumor 8T, a gene previously identified with a *T2/Onc* insertion in mouse tumor samples [11]. We also compared the zebrafish tumor integration gene list with the Cancer Genome Project Cancer Gene Census (http://www.sanger.ac.uk/genetics/CGP/Census/) [46]. This revealed that eleven of the zebrafish genes (11/149; 7.4%) have human homologs that are causally implicated in various cancers (Table 6): *CBFB*, *CBL*, *EXT1*, *GATA2*, *MSI2*, *NCOA2*, *NCOA4*, *PBX1*, *PRDX5*, *SMARCB1*, and *TSHR* (Table 6). We performed the same analyses using the integration data from all control samples. Twelve of the 602 genes (2%) from the control samples were represented in the Cancer Gene Census list (*BRD3*, *CBFB*, *CRLF2*, *ETV1*, *GPHN*, *FANCG*, *JAZF1*, *KTN1*, *MAF*, *NFKB2*, *NRAS*, *SFPQ*), and nineteen (3.2%) were present in the mouse RTCGD database (*Cnr2*, *Dlst*, *Fancg*, *Ghr*, *Igf1r*, *Inadl*, *Jazf1*, *Map2K5*, *Nfkb2*, *Nras*, *Nrxn2*, *Ppap2b*, *Ppp1r14b*, *Prlr*, *Rras2*, *Rtn4ipl*, *Tmem86a*, *Vac14*, *Zdhhc18*). The observed frequencies of integration into known cancer genes are significantly higher in the tumor samples than expected by chance (chi-square test *P*<0.002 and *P*<0.02 when comparing representation in Cancer Census List and RTCGD, respectively). Together, the results indicate the zebrafish tumors induced by *T2/OncZ* mutagenesis have an increased frequency of mutations in the homolog of known human and mouse cancer genes. This analysis validates our mutagenesis approach and provides additional comparative support for the involvement of these genes in human cancer.

**Table 6:** Comparison of zebrafish and human and mouse cancer genes.

Zebrafish Gene*	Human Gene#	COSMIC Cancer Gene Census%	Mouse RTCGD Cancer Gene∧
*brd2b*	*BRD2*	-	X
*cbfb*	*CBFB*	X	-
*cbl*	*CBL*	X	-
*cirbp*	*CIRBP*	-	X
*ext1c*	*EXT1*	X	-
*fgf8a*	*FGF8*	-	X
*gata2a*	*GATA2*	X	-
*zgc:162976*	*HEXIM1*	-	X
*zgc:172137*	*MAP2K5*	-	X
*mmp14b*	*MMP14*	-	X
*msi2b*	*MSI2*	X	-
*ncoa2*	*NCOA2*	X	X
*zgc:55307*	*NCOA4*	X	-
*pbx1a*	*PBX1* $	X	-
*prdx5*	*PRDX5*	X	-
*slc30a5*	*SLC30A5*	-	X
*smarcb1b*	*SMARCB1*	X	-
*snapc3*	*SNAPC3*	-	X
*si:dkey-76p14.4*	*SOX4*	-	X
*sox5*	*SOX5*	-	X
*LOC560609*	*TSHR*	X	-

*Zebrafish genes with *T2/OncZ* insertion in tumor tissue in the present study.

# Human Gene: *BRD2*, bromodomain containing 2; *CBFB*, core-binding factor, beta subunit, *CBL*, Cas-Br-M (murine) ecotropic retroviral transforming sequence; *EXT1*, exostosin 1; *FGF8*, fibroblast growth factor 8 (androgen-induced); *GATA2*, GATA binding protein 2; *HEXIM1*, hexamethylene bis-acetamide inducible 1; *MAP2K5*, mitogen-activated protein kinase kinase 5; *MMP14*, matrix metallopeptidase 14 (membrane-inserted); *MSI2*, musashi homolog 2 (Drosophila); *NCOA2*, nuclear receptor coactivator 2; *NCOA4*, nuclear receptor coactivator 4; *PBX1*, pre-B-cell leukemia homeobox 1; *PRDX5*, peroxiredoxin 5; *SLC30A5*, solute carrier family 30 (zinc transporter), member 5; *SMARCB1*, SWI/SNF related, matrix associated, actin dependent regulator of chromatin, subfamily b, member 1; *SNAPC3*, small nuclear RNA activating complex, polypeptide 3, 50 kDa; *SOX4*, SRY (sex determining region Y)-box 4; *SOX5*, SRY (sex determining region Y)-box 5; *TSHR*, thyroid stimulating hormone receptor.

% Gene identified as a human Cancer Gene in the Cancer Gene Census List.

∧ Gene identified as a CIS in mouse tumor tissues by retroviral or *T2/Onc* transposon insertion. X, present in Human Cancer Gene Census or mouse RTCGD: -, absent.

^$^ *PBX1* is mutated by translocation in human pre B-ALL, myoepithelioma.

## Discussion

The SB *T2/OncZ* system presented in this study provides a robust strategy for insertional mutagenesis in somatic tissues in zebrafish. The results demonstrate that transient or constitutive sources of *SB11* transposase effectively mobilize transposition from a stable *T2/OncZ* concatemer followed by genome-wide re-integration. *T2/OncZ* re-integration was randomly distributed across the genome and evenly distributed along each chromosome. The exceptions were a high number of re-integration events on chromosome 16 in concatemer line *Tg*(*T2/OncZ*, ß*-actin:RFP*)*^is6^*, and on chromosome 5 in line *Tg*(*T2/OncZ*, ß*-actin:RFP*)*^is7^*, which indicates linkage of the concatemer to these chromosomes.

The somatic mutagenesis strategy described here results in highly mosaic tissues with respect to transposon integration sites. In each sample there was at least 1 integration event per Mb on each chromosome, and in some samples the number was as high as 6. Variation in the average number of integrations could represent differences in the density of genes along each chromosome, since the plotted data represents integration sites that map to unique locations in the genome. Remarkably, we were able to detect multiple independent integration events in 21–28% of tagged genes from control fish when the constitutive ß*-actin:SB11* transgene was used as the transposase source. Multiple integration sites within a gene were also detected in 11–16% of tagged genes in DNA samples from fish that were injected with *SB11* mRNA. Intragenic excision and re-integration has previously been reported in mouse *T2/Onc* tumors [10], [11], [17], [18] and was interpreted to represent the selection of integrations that create mutations favorable for tumor cell survival and maintenance. The data presented here suggest that similar events occur with the zebrafish *T2/OncZ* system, not only in tumor tissues, but in control tissues as well. The ß*-actin:SB11* transgene appears to provide a constant level of transposase. This is evident in the absence of a large number of re-integration events surrounding the concatemer linkage site on chromosome 16 in tumors from double transgenic *Tg*(ß*-actin:SB11*)*^is8^*; *Tg*(*T2/OncZ*, ß*-actin:RFP*)*^is6^* fish aged 1–1.5 years and the increased frequency of multiple transposon insertions in single genes. The data are consistent with continuous mobilization and dispersal of *T2/OncZ* transposons across the genome as the fish age.

Given the robust transposition and transposon re-integration we observed in somatic tissues, the transgenic *T2/OncZ* system could be an effective tool for germline mutagenesis screens. *SB* was first shown to promote germline transposition and transgenesis in mice [47], [48] and was further developed as a germline mutagenesis tool [47], [49]. In zebrafish *SB* has been used for transgenesis and germline mutagenesis screens by injection of transposase RNA and transposon DNA into the embryo [50], [51], [52], [53]. Similar strategies for *SB*-mediated transgenesis in the germline have also been demonstrated in medaka [54] and *Xenopus* [55]. We show in the current study that the *T2/OncZ* system promotes a high level of transposition and genome-wide integration. Germline mutagenesis in the mouse starting with transgene concatemers has previously been shown for both the *T2/Onc* transposon [41] and a gene trap *T2* vector [56]. Adopting the *T2/OncZ* system for germline mutagenesis might increase the efficiency of insertional mutagenesis screens compared to previous transgenic methods in zebrafish [57].

Introducing a transient source of transposase into the high copy number *T2/OncZ* concatemer line by RNA injection resulted in a tumor frequency of 10%. The occurrence of tumors was surprising, given that transposase expression is transient and the embryo is mosaic for integration sites in individual cells. Although 2 out 3 double transgenic *Tg*(ß*-actin:SB11*)*^is8^*; *Tg*(*T2/OncZ*, ß*-actin:RFP*)*^is6^* developed tumors, the small number of fish that survived to adulthood prevents an estimate of the true frequency of tumor induction using constitutive transposase. We are currently testing an alternative *Tg*(ß*-actin:SB11*) line to optimize SB11 expression levels, as well as additional tumor suppressor genetic backgrounds, in order to increase the frequency of tumor induction.

This study demonstrates the capability of the *Sleeping Beauty T2/OncZ* system for genome-wide insertional mutagenesis in somatic tissues in zebrafish and the potential for identifying novel cancer genes. The comparison of *T2/OncZ* integration sites in the five zebrafish tumors to the human Cancer Gene Census and mouse Retroviral and Transposon Cancer Genome Database revealed overlapping genes. Three of the five tumors had a *T2/OncZ* insertion in one gene (*CBL*, *TSHR*, or *SMARCB1*) known to be highly mutated in human cancers [46]. While these insertions could be interpreted as the activating mutations responsible for initiating cellular transformation and tumor progression, similar insertions from multiple tumors are necessary to support that conclusion. This comparative approach among zebrafish, mouse and human provides additional evidence in support of putative cancer genes and the conservation of genetic pathways mutated in cancer.

Future studies examining integration events in large numbers of zebrafish tumors will be necessary to identify novel cancer genes as has been done previously in mouse *SB*-induced cancer models [10], [11], [17], [18]. Modification of the system using alternative *T2/OncZ* vectors, or combining transposase injection and transgenic sources, could increase tumor frequency. However, the ability to scale up the mutagenesis strategy in order to recover significant numbers of tumor samples is straightforward using zebrafish. For a standard zebrafish laboratory it is feasible to inject 500 embryos, or recover 500 double transgenic fish, and raise the fish to adulthood. One important future use of the *T2/OncZ* system is to combine it with other zebrafish models of human cancers to screen for enhancers of tumor onset, progression and possibly metastasis. These studies will provide a novel strategy for elucidating the genetics pathways that drive cellular transformation and tumorigenesis in human cancer.

## Methods

### Ethical Statement

Animals were reared and euthanized according to protocol #06-D-029-A approved by the Iowa State University IACUC Committee. All efforts were made to minimize suffering.

### Zebrafish strains

Zebrafish were reared and housed in an AHAB system (Aquatic Ecosystems, Inc.) and kept under a 14 hour light/10 hour dark photoperiod at 27°C. Wild type zebrafish were obtained from 5-D Tropical Inc. (Florida). WIK, TU, and the *tp53^M214K^* lines were obtained from the Zebrafish International Research Center (ZIRC).

### Isolation of transgenic Tg(*T2/OncZ*, ß-actin:RFP) concatemer lines

The SB *T2/OncZ* transposon was built by modifying the previously characterized *T2/Onc* vector [10] by inserting the carp ß*-actin* promoter [33] (detailed cloning steps are provided in Methods S1. To isolate zebrafish lines carrying *T2/OncZ* concatemer arrays linear DNA fragments containing *T2/OncZ* and the ß*-actin:RFP* reporter cassette were co-injected into embryos. The *pT2/OncZ* vector was digested *Pvu*I and *BsaX*I to release a ∼4.9 Kb fragment containing the transposon flanked by 160 bp and 280 bp of vector sequence at the 5′ and 3′ ends, respectively. The 4.9 Kb *T2/OncZ* fragment was mixed at a 2∶1 ratio with a 3.1 Kb fragment containing a ß*-actin:RFP* reporter cassette to a final concentration of 250 pg/nl. The DNA was diluted 1× in Danio buffer and 125–250 pg injected into 1-cell WIK or wild type embryos. Founders were raised to adulthood and screened by outcrossing with WIK or wild type and examining F1 embryos for ubiquitous RFP expression at 24 hpf. Out of 54 founders screened, seven independent F1 concatemer lines were recovered. *Tg*(*T2/oncZ*, ß*-actin:RFP*)*^is6^* (>500 copy number) and *Tg*(*T2/OncZ*, ß*-actin:RFP*)*^is7^* (75 copy number) were kept and the remaining 5 lines were not maintained.

Genomic Southern blotting, Western blotting, transposon excision PCR assay, and isolation of transposase mRNA are described in Methods S1.

### Isolation of *SB11* transposase transgenic lines by *Tol2*-mediated transgenesis

Details of the molecular construction of *Tol2<*ß*-actin:SB11*, *cmlc2:GFP>* are provided in Methods S1. To isolate SB11 transposase expressing transgenic fish, 50 pg Tol2 mRNA and 100 pg *Tol2<*ß*-actin:SB11*, *cmlc2:GFP>* plasmid was coinjected into the 1-cell stage WIK embryos. At 24 hpf injected embryos were screened for mosaic GFP expression in the heart and positive embryos were selected and raised to adulthood. Adult founders were outcrossed with wild type and the F1 embryos screened for GFP expression in the heart. Nine independent F1 *Tg*(*Tol2<*ß*-actin:SB11*, *cmlc2:GFP>*) lines were recovered after screening 22 F0 founder adults. Lines *Tg*(*Tol2<*ß*-actin:SB11*, *cmlc2:GFP>*)*^is8^* and *Tg*(*Tol2<*ß*-actin:SB11*, *cmlc2:GFP>*)*^is9^* were maintained.

### Somatic Mutagenesis Strategies

Heterozygous transgenic *Tg*(*T2/OncZ*, ß*-actin:RFP*) adult males were crossed to heterozygous *Tg*(*Tol2<*ß*-actin:SB11*, *cmlc2:GFP>*) females. Embryos were collected and aged to 24 hpf or 48 hpf and sorted into 4 progeny classes based on ubiquitous RFP expression and heart-specific GFP expression. Approximately, 50 individuals in each progeny classes were reared in separate tanks and examined for macroscopic tumors before sacrificing and harvesting tissues for DNA isolation and pathology. For transposase mRNA injections, *Tg*(*T2/OncZ*, ß*-actin:RFP*) males were outcrossed to WIK females and ∼50 pg of *SB11* mRNA injected into 1-cell stage embryos. At 24 hpf embryos were separated into RFP+ and RFP- classes and reared in separate tanks.

### Histopathology

Tissue and tumors were dissected from anesthetized adults and fixed in 10% buffered Formalin (Fisher). All tissues were embedded in paraffin and processed for routine sectioning (4 um) and hematoxylin and eosin staining (H&E) at the Histopathology Laboratory, Department of Veterinary Pathology, in the College of Veterinary Medicine at Iowa State University.

### Identification of transposon integration sites

Sequences flanking transposon integration sites were PCR amplified using a previously described adapter ligation-mediated PCR protocol [16] that was modified for use with the Illumina/Solexa Genome Analyzer II. Bar-coded primers used for amplification are listed in Table 3 and additional methods are found in Methods S1.

### Computational Analyses

Illumina sequences were trimmed to remove primer and transposon sequences and mapped to the zebrafish genome assembly v8 using BLAT. Graphical plots of transposon distribution and density across the genome were created using the R program. Chi-square tests were performed at http://people.ku.edu/~preacher/chisq/chisq.htm [58] and http://www.graphpad.com/quickcalcs/chisquared1.cfm?Format=C.

### Comparative Genomic and Network Analyses

Filtered datasets of integration sites were analyzed for cross-contamination between samples processed on the same day and sequenced in the same Illumina run. The identical location for 6 integration sites in the tumor samples was also present in one or more control samples. The corresponding genes were removed from the tagged gene list before performing comparative genomic analyses.

## Supporting Information

Dataset S1 **Tabs are provided for all** ***T2/OncZ*** **integration sites from SB11 mRNA injected tumor and control zebrafish (all sites SB11 injected), and all** ***T2/OncZ*** **integration sites from tumor and control zebrafish with** ***Tg*** **(** ***Tol2<*** **ß** ***-actin:SB11*** **,** ***cmlc2:GFP>*** **) (all sites BaSB11 transgene).**
(XLS)

Dataset S2 **Tabs are provided for all** ***T2/OncZ*** **integration sites remaining in control samples and tumor samples after filtering the lists to remove sites that fall below two standard deviations above the mean.**
(XLS)

Methods S1 (DOC)
