# Supplementary material for: Somatic Mutagenesis with a Sleeping Beauty Transposon System Leads to Solid Tumor Formation in Zebrafish
Source: PLoS One. 2011 Apr 21;6(4):e18826. doi: 10.1371/journal.pone.0018826 (PMC3080878; doi:10.1371/journal.pone.0018826)
Supplement: Methods S1 — (DOC) [file pone.0018826.s003.doc]

**Supporting Information Methods S1**

***Molecular constructs***

Constructs and transposon vectors were built using standard PCR-based molecular cloning techniques. Proofreading polymerase *Pfu* (Invitrogen) was used in all PCR reactions, and the sequences for each construct were confirmed using Sanger sequencing.

***SB T2/OncZ transposon***

The *T2/OncZ* transposon was built by modifying the previously characterized *T2/Onc* vector [10] with the addition of the carp ß*-actin* promoter [33]. The truncated 2476 bp carp ß*-actin* promoter fragment contains 1137 bp of 5’ promoter sequence, the 92 bp non-coding exon 1, and 1247 bp of the first intron, which lacks the splice acceptor sequences at the 3’ end. The truncated carp ß*-actin* promoter was cloned in between the MSCV 5’ LTR and SV40 pA in *T2/Onc*. First, the entire plasmid containing *T2/Onc* was PCR amplified with primers carrying *Spe*I and *Nco*I restriction enzyme sites. The *Spe*I containing reverse primer (5’ TAA ACT AGT TAH CTT GCC AAA CCT ACA GGT G 3’) sits just 5’ to the start of the MSCV 5’ LTR in *pT2Onc* at postion 628. The *Nco*I containing forward primer (5’ ATA CCA TGG AAG ATA CAT TGA TGA GTT TGG AC 3’) sits just 3’ to the MSCV 5’ LTR in *pT2/Onc* at position 1194. Second, the ~2.5 Kb carp ß*-actin* promoter was amplified from the *pFRM2/Red* vector (Dr. Steve Ekker, Mayo Clinic, Rochester, MN) using a forward *Spe*I containing primer (5’ TAT ACT AGT TTA GAC CTT CTT ACT TTT GGG GA 3’) and reverse *Nco*I containing primer (5’ ATT GAA TTC CCA TGG AGT GCA CAG CTG TCA GGG AA 3’). The amplified carp ß*-actin* promoter is missing 35 bp at the 3’ end of intron 1 that contains splice acceptor sequences. The amplified *T2/Onc* vector and carp *β-actin* promoter were digested with *Spe*I and *Nco*I enzymes, ligated, and cloned using standard cloning methods. Third, the MSCV 5’LTR was cloned back into the modified *T2/Onc* transposon. The MSCV 5’ LTR was amplified with forward and reverse primers containing *Spe*I restriction sites (F 5’ TAT ACT AGT GCT TAA GTA ACG CCA TTT TGC AAG 3’; R 5’ AAT ACT AGT GCA TGC GGG CGA CGC AGT CTA TCG GA 3’) and cloned into the *Spe*I site in the modified *T2/Onc* to make the final vector *pT2/OncZ*.

The RFP reporter cassette used for transgenesis was assembled in *pBlueScript* (Stratagene) by cloning a shortened version of the carp *β-actin* promoter in front of the *DsRed2* cDNA (Clontech). The AFP 3’UTR was subcloned from *pFRM2/RED* into the construct behind *DsRed2*.

***Ubiquitous SB11 transposase vectors***

The carp ß*-actin* promoter:*SB11* cDNA trangene was assembled inside a mini*Tol2* vector [38]. First, the mini*Tol2* inverted repeat arms and vector backbone were amplified from the plasmid *pGemT-Tol2* [38] using a *Not*I-containing reverse primer (5’ ATT TAG CGG CCG CAA CTG GGC ATG AGC GCA ATT C 3’) and *Cla*I-containing forward primer (5’ AAT ATA ATC GAT AAA CAAGAA TCT CTA GTT TTC TTT C 3’). Second, an oligo linker containing multiple cloning sites was synthesized and ligated into the *Not*1 and *Cla*I sites of the amplified mini*Tol2* vector to create the plasmid vector *pTol2lMCS2*. Third, a GFP reporter cassette was assembled by PCR amplification and stepwise cloning into *pBlueScript*. The *cardiac myosin light chain* 2 (*cmlc2*) promoter was amplified and from the plasmid *pDestV* (Dr. Chi-Bin Chien, University of Utah) and assembled with the EGFP cDNA (Clonetech) and the zebrafish ß*-actin* 3’ UTR (Dr. Darius Balciunas, Temple University). A *Sac*II-*Kpn*I fragment containing the *cmlc2:EGFP* cassette was then subcloned into *Sac*II-*Kpn*I-digested *pTol2MCS2*. *pTol2<*ß*−actin:SB11; cmlc2:EGFP>* was constructed by first assembling a carp ß*-actin* promoter:*SB11* cDNA:zf ß*-actin* 3’UTR cassette in *pBluescript* and subcloning a *Not*I-*Xma*I fragment containing the cassette into a *Not*I-*Xma*I digested *pTol2MCS2*, 5’ to the *cmlc2:EGFP* cassette.

***Synthesis of transposase mRNAs***

*SB11* [37] and *Tol2* [38] transposase capped mRNAs were synthesized *in vitro* from 1 ug of linearized *pT3TSSB11* and *pT3TS-Tol2* templates using the mMESSAGE mMACHINE High Yield Capped RNA transcription kit (Ambion, Cat. No. AM1348). mRNAs were precipitated using LiCl and resuspended in water.

***Genomic Southerns***

To isolate genomic DNA for Southern analysis, adult zebrafish were anesthetized and quick frozen in liquid nitrogen, ground in a mortar and pestle, and DNA extracted using the Qiagen Blood and Cell Culture Maxi Kit (Qiagen, Cat. No. 13362). Approximately 10 ug of DNA was digested with appropriate enzymes. The *pT2/OncZ* plasmid was digested and loaded on the gel for the copy number control. The Southern blot was prepared using a Whatman Turboblotter Rapid Downward Transfer System (ISC BioExpress, Cat. No. F-3131-14). Probes were synthesized using the PCR-DIG Probe Synthesis Kit (Roche, Cat. No. 11636090910). Hybridization and detection were performed with DIG Easy Hyb Granules/Wash and Block Buffer Set and CSPD (Roche, Cat. Nos. 11796895001, 11585762001, 11655884001). Images were captured on a BioRad ChemiDoc XRS system and band density determined using ChemiDoc software.

***Transposon excision PCR assay***

Individual 5 dpf zebrafish larvae were placed into 25 ul Tris-EDTA and frozen at -20^o^C. Proteinase K was added to 200 ug/ml and the larvae incubated at 55^o^C for at least 5 hours. Proteinase K was inactivated by incubation for 15 minutes at 98^o^C. 2 ul was used as template for PCR reactions using GoTaq Green 2X with pairs of the following primers as described in the results section: SBprimer1 5’ ATG TGC TGC AAG GCG ATT AAG TTG 3’; SBprimer2 5’ ATC AAG CTT CTA AAG CCA TGA CAT C 3’; SBpimer3 5’ ATC TAG CTT GTG GAA GGC TAC TCG 3’; and SBprimer4 5’ TGA GCG GAT AAC AAT TTC ACA CAG G 3’.

***Western Blotting***

Embryos from transgenic females outcrossed with wild type males were collected at the 1-cell stage. To heat shock samples, embryos were aged to early gastrula stage (5 hpf) and placed in 37^o^C water bath for 2 hours. For SDS-PAGE and western analysis embryos were harvested in sample buffer and equivalent numbers of embryos loaded in each lane. The anti-SB monoclonal (R&D Systems, Minneapolis, MN) and anti-β-actin monoclonal (Sigma) primary antibodies were used at a 1:1000 dilution. Blots were probed with HRP-conjugated secondary antibody (Zymed) diluted 1:2000, developed with ECL SuperSignal chemiluminescence reagents (Pierce), and imaged on a BioRad ChemiDoc XRS system.

***Identification of transposon integration sites***

Sequences flanking transposon integration sites were PCR amplified using a previously described adapter ligation-mediated PCR protocol [16] that was modified for Solexa sequencing on a Genome Analyzer II. DNA isolated from *Tg(T2/OncZ,* ß*-actin:RFP)* tumors or control tissue was digested separately with *Bfa*I and *Nla*III to amplify junction fragments from the left and right side of the transposon, respectively. Each sample was ligated with an adaptor and amplified with a unique bar-coded primer (Table 3) and adaptor-specific primer. A secondary digest with *BamH*I was carried out before PCR to eliminate amplification of transposons within the concatemer. A portion of each library was shotgun-cloned and submitted for standard Sanger sequencing to confirm the PCR products were integration site junction fragments. For massively parallel sequencing, twelve samples were pooled and run in a single lane on an Illumina/Solexa Genome Analyzer II at the Iowa State University DNA Facility (Ames, IA).
